# Failure of *Burkholderia pseudomallei* to Grow in an Automated Blood Culture System

**DOI:** 10.4269/ajtmh.14-0018

**Published:** 2014-12-03

**Authors:** Nittaya Teerawattanasook, Direk Limmathurotsakul, Nicholas P. J. Day, Vanaporn Wuthiekanun

**Affiliations:** Medical Technology Department, Sappasithiprasong Hospital, Ubon Ratchathani, Thailand; Mahidol-Oxford Tropical Medicine Research Unit, and Department of Tropical Hygiene, Faculty of Tropical Medicine, Mahidol University, Bangkok, Thailand; Centre for Tropical Medicine and Global Health, Nuffield Department of Medicine, University of Oxford, Oxford, United Kingdom

## Abstract

We compared the organisms isolated from 30,210 pairs of blood culture bottles by using BacT/Alert system and the conventional system. Overall, 2,575 (8.5%) specimens were culture positive for pathogenic organisms. The sensitivity for detection of pathogenic organisms with the BACT/Alert system (85.6%, 2,203 of 2,575) was significantly higher than that with the conventional method (74.1%, 1,908 of 2,575; *P* < 0.0001). However, *Burkholderia pseudomallei* was isolated less often with the BacT/ALERT system (73.5%, 328 of 446) than with the conventional system (90.3%, 403 of 446; *P* < 0.0001). This finding suggests that use of the conventional culture method in conjunction with the BacT/Alert system may improve the isolation rate for *B. pseudomallei* in melioidosis-endemic areas.

The Gram-negative bacillus *Burkholderia pseudomallei* is a Tier 1 select agent and the cause of melioidosis.^1^ The disease accounts for 20% of all community-acquired septicemias in northeastern Thailand,^2^ where melioidosis is the third most frequent cause of death from infectious diseases.^3^ Melioidosis is notoriously difficult to cure despite appropriate antimicrobial therapy and has a case-fatality rate of up to 43%.^3^ More than half of all melioidosis patients are bacteremic, and positive blood cultures for *B. pseudomallei* obtained at hospital admission and/or during hospitalization are strong prognostic markers for death.^4^

Although the automated blood culture system (BacT/Alert) is convenient and currently used in many laboratories in provincial hospitals in Thailand, it is unclear whether its sensitivity for the detection of pathogens is similar to that obtained using a conventional low tech system still commonly used in small hospitals in resource-limited settings.

In a retrospective study conducted during January 1, 2009–July 31, 2011 as part of routine patient care at Sappasithiprasong Hospital, a 1,000-bed tertiary-care hospital in northeastern Thailand, we compared the organisms isolated from more than 30,000 pairs of blood culture bottles by using the BacT/Alert system and the conventional system.

In conventional system, the culture medium is made in-house, blood culture bottles are incubated in a conventional incubator, and bacterial detection is made by direct visualization with or without regular sub-culture. During the study period, two 5-mL blood samples were regularly obtained from each patient 10–15 minutes apart. The first 5 mL of blood was inoculated into a 40 mL culture media BacT/Alert SA bottle (catalog no. 259789; bioMérieux, Durham, NC). The second 5 mL of blood was inoculated into an in-house bottle containing 40 mL of broth, which consisted of 37 g of brain heart infusion medium broth (catalogue no, 211059; Becton Dickinson, Franklin Lakes, NJ) and 0.25 g of sodium polyanatholesulfonate (catalog no.1000907362; Sigma, St. Louis, MO), in 1 liter of purified water. BacT/Alert bottles were incubated in the BacT/Alert automated blood culture system (bioMérieux) at 35°C for 7 days, and in-house bottles were incubated aerobically in a normal incubator at 35°C for 7 days. Examination of BacT/Alert bottles was done according to the directions provided by the manufacturer, and positive cultures were indicated on the computer screen accompanied by a beeping sound. Positive cultures in the in-house bottles were detected by direct visualization of cloudy broth.

Positive bottles from both systems were sub-cultured by using airway needles (bioMérieux) to place approximately 15–20 μL of the culture on chocolate agar, blood agar, and eosin-methylene blue agar. In addition, all in-house bottles were routinely sub-cultured onto blood agar on day 2 of incubation, and all in-house and BacT/Alert bottles were routinely sub-cultured onto blood agar on day 7 of incubation. Blood agar and eosin-methylene blue agar plates were incubated aerobically at 35°C and inspected at 24 hours. Chocolate agar plates were incubated in an atmosphere of 5% CO_2_ at 35°C and were inspected at 48 hours. Bacterial or fungal colonies that grew on culture plates were identified by using standard biochemical tests and colonies of presumptive *B. pseudomallei* were identified by typical colony morphology on Ashdown agar, resistance to gentamicin and colistin, and a positive result for a highly specific latex agglutination test, as described.^5^,^6^

A total of 30,210 pairs of blood culture bottles were collected during the study period (10,208, 12,574 and 7,428 in 2009, 2010 and 2011, respectively). Overall, 2,575 (8.5%) specimens were culture positive for pathogenic organisms. A total of 1,536 (59.7%) grew with both methods, 667 (25.9%) grew only with the BacT/Alert system, and 372 (14.4%) grew only with the conventional method.

The pathogenic organisms isolated were gram-negative bacteria (68.1%), gram-positive bacteria (20.0%), fungi (9.7%) and polymicrobial organisms (2.2%). The most common pathogens were *Escherichia coli* (18.9%, 486 of 2,575), *B. pseudomallei* (17.3%, 446 of 2,575), *Klebsiella* species (10.3%, 266 of 2,575), *Staphylococcus aureus* (8.5%, 219 of 2,575), and *Pseudomonas* spp. (8.0%, 207 of 2,575) (Table 1).

In general, the sensitivity for detection of pathogenic organisms with the BACT/Alert system (85.6%, 2,203 of 2,575) was significantly higher than for the conventional method (74.1%, 1,908 of 2,575; *P* < 0.0001, by McNemar's exact test). However, *B. pseudomallei* was isolated less often with the BacT/ALERT system (73.5%, 328 of 446) than with the conventional system (90.3%, 403 of 446; *P* < 0.0001, by McNemar's exact test), and 118 (27%) *B. pseudomallei* bacteremias would not have been detected over this two-year period if only the BacT/ALERT system had been used (Table 1).

The median time to positivity of *B. pseudomallei* with the BacT/ALERT system was much faster than with the conventional system (1 day versus 2 days; *P* < 0.0001, by Wilcoxon signed-rank test) (Figure 1). The result of a less sensitive detection of conventional system during the first few days might not have been caused by the medium used but by a difference of the technique used to detect the positivity between the conventional system (observation of cloudy broth on day one and routine subculture of all bottles on day two) and the BacT/ALERT system. However, 88 (19.7%) of 446 were positive from sub-culture on day 7 with the conventional system and only 2 (0.5%) of 446 were positive with the BacT/ALERT system (Figure 1).

**Figure 1.**
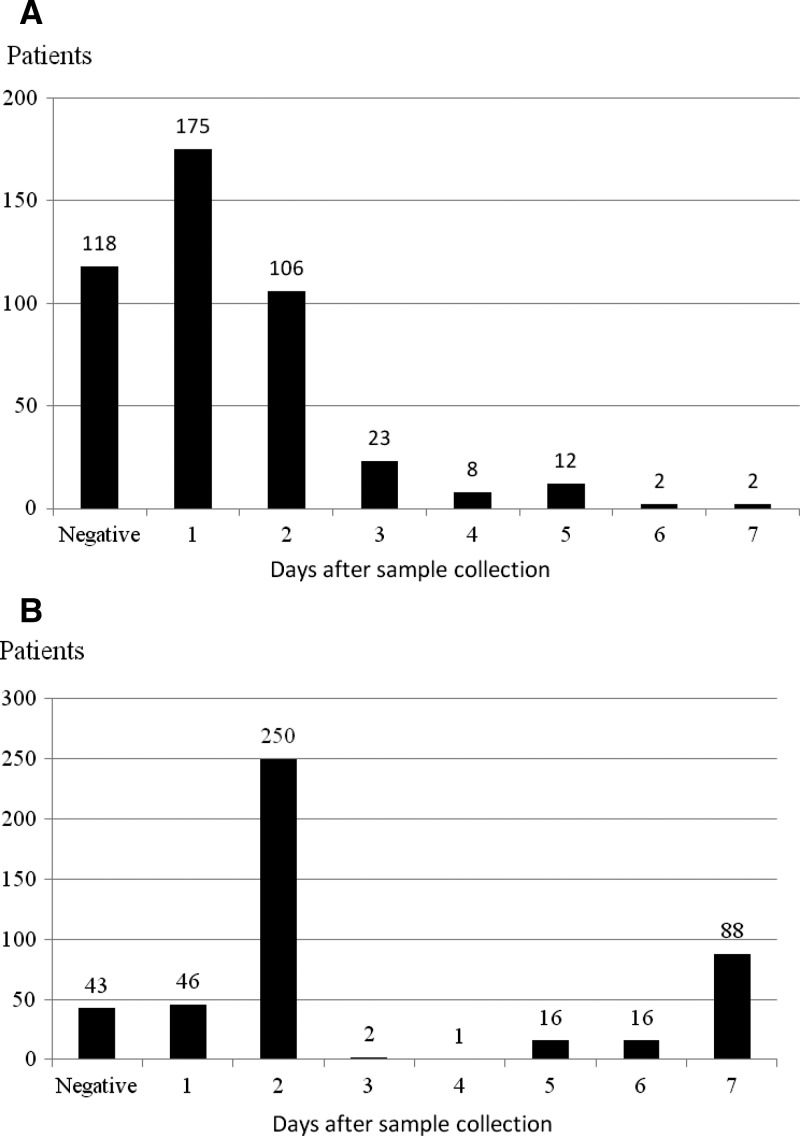
Time to blood culture positivity for 446 patients whose blood culture was positive for *Burkholderia pseudomallei* with either the **A**, BacT/Alert system (1A) or **B**, the conventional system, northeastern Thailand.

In this study, we demonstrated that the BacT/Alert system is more sensitive than the conventional system for culture of most pathogenic organisms, but not for *B. pseudomallei*. Diagnosis of melioidosis is based on culture positivity for *B. pseudomallei.* Low sensitivity of the BacT/Alert system may lead to the misdiagnosis of a number of melioidosis patients and an underestimation of the prevalence of melioidosis if the conventional method is not used.

Although *B. pseudomallei* isolates grow faster in the BacT/Alert system, we found that a higher proportion of *B. pseudomallei* isolates were detected with the conventional system, especially with the sub-culture on day 7. The BacT/Alert medium has a different nutrient composition compared with that of the conventional medium, which may explain the difference. *Burkholderia pseudomallei* is a slow-growing bacterium and the nutrient composition of the conventional medium may support this slow growth better. Further studies are required to evaluate this phenomenon. This study suggests that using the conventional culture method with brain heart infusion broth in conjunction with BacT/Alert system with tryptic soy broth may improve isolation of *B. pseudomallei* in melioidosis-endemic areas.

## Figures and Tables

**Table 1:** Pathogenic organisms isolated with BacT/Alert system and the conventional system at Sappasithiprasong Hospital, northeastern Thailand*

Organism	No. positive samples (%), n = 2,575)	No. positive samples			*P*†
		BacT/Alert and conventional systems	BacT/Alert system	Conventional system	
*Escherichia coli*	486 (18.9)	321	117	48	< 0.0001
*Burkhloderia pseudomallei*	446 (17.3)	285	43	118	< 0.0001
*Klebsiella* spp.	266 (10.3)	193	45	28	0.06
*Staphylococcus aureus*	219 (8.5)	160	36	23	0.12
*Pseudomonas* spp.	207 (8.0)	54	99	54	0.0004
Other organisms	894 (34.7)	482	327	101	< 0.0001
Polymicrobial infections	57 (2.2)	41	NA	NA	NA
Overall	2,575 (8.5)	1536	667	372	< 0.0001

* NA = not applicable.

† By McNemar's exact test.
